# Stimulating the Healthy Brain to Investigate Neural Correlates of Motor Preparation: A Systematic Review

**DOI:** 10.1155/2018/5846096

**Published:** 2018-02-04

**Authors:** Cécilia Neige, Hugo Massé-Alarie, Catherine Mercier

**Affiliations:** ^1^Center for Interdisciplinary Research in Rehabilitation and Social Integration, Québec, QC, Canada; ^2^Department of Rehabilitation, Laval University, Québec, QC, Canada

## Abstract

**Objective:**

Noninvasive brain stimulation techniques can be used to selectively increase or decrease the excitability of a cortical region, providing a unique opportunity to assess the causal contribution of that region to the process being assessed. The objective of this paper is to systematically examine studies investigating changes in reaction time induced by noninvasive brain stimulation in healthy participants during movement preparation.

**Methods:**

A systematic review of the literature was performed in the PubMed, MEDLINE, EMBASE, PsycINFO, and Web of science databases. A combination of keywords related to motor preparation, associated behavioral outcomes, and noninvasive brain stimulation methods was used.

**Results:**

Twenty-seven studies were included, and systematic data extraction and quality assessment were performed. Reaction time results were transformed in standardised mean difference and graphically pooled in forest plots depending on the targeted cortical area and the type of stimulation.

**Conclusions:**

Despite methodological heterogeneity among studies, results support a functional implication of five cortical regions (dorsolateral prefrontal cortex, posterior parietal cortex, supplementary motor area, dorsal premotor cortex, and primary motor cortex), integrated into a frontoparietal network, in various components of motor preparation ranging from attentional to motor aspects.

## 1. Introduction

The execution of a voluntary movement is preceded by a series of complex and interacting processes that occur in multiple brain areas. In 2015, Wong et al. [1] proposed a sequential categorization of these processes that operate from perception to movement onset. The first step, mainly perceptive and attentional, consists in identifying and then selecting the target of interest to create a “motor goal.” The second step includes sensorimotor processes and implies organization and specification of neuromotor activity patterns necessary to execute the movement, for example, the spatial and temporal timing of contraction of the selected muscles to achieve the motor goal.

Seminal work performed in 1980 by Rosenbaum [2] using a precued reaction time (RT) paradigm provided behavioral evidence of the existence of motor preparation. In this paradigm, an informative cue (the warning signal) concerning a forthcoming movement was given to the participant in advance of an imperative cue (the response signal) indicating that the movement can be executed. Informative warning signal on the forthcoming movement (e.g., the side to be used or distance of the target) leads to a shorter RT (the time between the response signal and motor response initiation). It represents a greater level of motor preparation [3] compared to a condition with an uninformative cue. Since then, RT has been considered the main outcome to assess the efficiency of processes that occur during motor preparation [4]. Over the last decades, various neurophysiological methods have been used to measure cortical activity before the execution of a voluntary externally cued movement in order to shed light on the mechanisms underlying movement preparation. A first approach consists in using single-pulse transcranial magnetic stimulation (TMS) applied over the primary motor cortex (M1) to probe corticospinal excitability during motor preparation (for a review, see [5]). It has been shown that many mechanisms including conflict processing, competition resolution, and impulse control occur during motor preparation (for recent reviews, see [6, 7]). Electroencephalography (EEG) can also be used to examine movement-related cortical potential. A slow negative cortical potential called the contingent negative variation (CNV) occurs prior to the onset of an externally cued movement [8] and is directly related to the level of motor preparation: larger CNV is associated with shorter RT [9]. In long motor preparation period (>1 s), CNV can be subdivided into two parts: the early period that reflects the perceptive and attentional processes and the late period which represents anticipation and motor preparation related to the forthcoming movement [10]. The supplementary motor area (SMA), premotor area, and parietal cortex have been reported to be involved in the generation of the CNV [11, 12]. Finally, studies using functional magnetic resonance imagery during a precued RT paradigm demonstrated a neural circuitry that includes several regions [13, 14]. Movement preparation activates a parieto-frontal network associated with the attentional and perceptive components of the motor preparation. For example, processing of spatial information about the target involves the parietal cortex whereas the motor goal and context of the forthcoming movement are encoded in the prefrontal cortex [15]. It has been notably suggested that the dorsolateral part of the prefrontal cortex plays a crucial role in attention and selective task-relevant information along motor preparation [16]. Moreover, several studies provided evidence that the M1 [17], SMA [18], and premotor cortex [19] are activated before the execution of the movement and may be involved in motor preparation processes.

Although neuroimaging techniques can provide information with a high spatial resolution about which brain regions are activated during motor preparation, their correlational approaches do not allow causal inference about the involvement of these regions. An alternative way to assess the functional role of cortical regions during motor preparation is to use noninvasive brain stimulation techniques such as repetitive transcranial magnetic stimulation (rTMS) or transcranial direct current stimulation (tDCS). With rTMS, a magnetic field induced by a coil placed over the scalp induces an electrical current in the targeted brain area that can be of sufficient magnitude to depolarize neurons transiently. With tDCS, a weak direct current (0.5–2.0 mA) circulates through the brain between a positive (cathode) and a negative (anode) electrode positioned on the scalp in order to modulate the ongoing neural activity (for a review, see [20]). Depending on stimulation parameters, these techniques can be used to selectively improve or disrupt processing in a specific cortical region in healthy participants [21, 22], then providing the opportunity to assess the causal contribution of the stimulated region or network to the process being assessed [23, 24]. Studies measuring the effect of these techniques applied to the M1 have shown that, generally speaking, low-frequency rTMS (≤1 Hz) and cathodal tDCS decrease cortical excitability, while high-frequency rTMS (≥5 Hz) and anodal tDCS increase the cortical excitability of the stimulated brain area [25–29]. Moreover, a patterned rTMS approach termed theta burst stimulation (TBS) can also transiently modify the excitability of the targeted brain area. It consists of triplets of pulses (trains) applied at 50 Hz repeating at 5 Hz and includes two different methods: continuous TBS (cTBS) consisting in TBS trains given without interruption for 20 s or 40 s which generally decreases excitability and intermittent TBS (iTBS) that involves 20 TBS trains of 2 s repeated every 10 s which generally increases excitability [30, 31]. It is however essential to keep in mind that most rTMS and tDCS studies do not directly verify whether their stimulation paradigm really induced inhibition or facilitation in the targeted area. Therefore, statements about inhibitory/excitatory effects remain hypothetical and are based on effects mainly observed for the M1 that are still considerably debated (for a recent review, see [32]).

The objective of this paper is to systematically examine studies that have addressed the causal contribution of the brain regions or networks that are believed to play a role in motor preparation. More specifically, this systematic review focuses on findings from noninvasive brain stimulation studies performed in healthy participants to probe the neural correlates of movement preparation, using changes in RT as the main outcome variable.

## 2. Methods

This systematic review was conducted in accordance with the PRISMA statement [33].

### 2.1. Data Sources

The development of the systematic search strategy was established with the help of a professional librarian. A systematic literature search was conducted using electronic databases PubMed, MEDLINE, EMBASE, PsycINFO, and Web of science and from the date of inception to May 9, 2017.

Relevant manuscripts were identified using a combination of keywords and medical subject headings (MeSH) related to motor preparation and associated behavioral or neurophysiological outcomes and noninvasive brain stimulation methods. A list of terms related to animal studies was excluded. No restriction about the year of publication was applied. The search strategy adapted for each database is provided in Supplementary Data 1. An additional hand search in the reference lists of relevant articles was performed.

Search results were exported to EndNote X6 (Clarivate Analytics), a reference management software, for automatic (and visually checked) duplicate removal. A preliminary screening of the title and abstract was performed by one of the authors in order to remove irrelevant articles. Then, the full text of potentially relevant papers was reviewed to determine their eligibility.

### 2.2. Inclusion Criteria

Studies were included if they met the following criteria: (1) published in peer-reviewed journals in English or French; (2) involved healthy participants aged 18–65 years (studies with participants over 65 years old excluded because motor preparation has been shown to be affected by age [34]; (3) assessed motor preparation of an externally cued voluntary movement of the upper limb (studies that assessed high level of cognitive planning only were excluded); (4) included a measurement of change in RT; (5) used a noninvasive brain stimulation offline paradigm (tDCS or rTMS) (i.e., stimulation was applied with the participant at rest, with pre-post measurements or comparison to a sham to control for nonspecific effects on RT [35, 36]); and (6) was a research paper including an original data set (case studies, conference abstracts, and reviews were excluded).

### 2.3. Data Extraction

Information collected from each study included the name of the first author and year of publication, number of participants (*n*), experimental design, task, type of stimulation, frequency, pattern, duration of the stimulation, intensity, technical aspects about the coil or electrodes, cortical area targeted by stimulation, and RT results. Mean and standard deviation of RT pre- and poststimulation were extracted for each paper when it was possible. When only the standard error (SE) was provided, the standard deviation (SD) was calculated using the formula SD = SE × √*n*. Plot digitizer software (2.6.8 version) was used to extract data from figures and graphs if it was not available in a numerical form. It has been shown that this software is faster with a greater interrater reliability compared to manual extraction using a ruler [37]. Finally, the authors were contacted when relevant data could not be retrieved in the article.

Meta-analyses on pre-post effect size for the single group are not recommended, mainly because outcomes obtained on pretest and posttest are not independent from each other [38]. However, the standardised mean difference (SMD) is still appropriate for the synthesis of studies assessing the same outcome in order to provide a visual summary of the results. Therefore, SMD for RT between pre- and poststimulation were calculated and graphically pooled in forest plots for each subgroup depending on the site and the type of stimulation, but no meta-analysis was performed on these data.

The SMD for the pre-post single group design studies were calculated using the following Becker's equation [39]:

(1) $$\begin{array}{c} {SMD}_{Becker\;\left(B\right)}=c\left(n-1\right)\frac{{mean}_{poststimulation}-{mean}_{prestimulation}}{standard\;{deviation}_{prestimulation}}, \end{array}$$

where *n* is the number of participants in the study and *c*(*n* − 1) = 1 − (3/(4(*n* − 1) − 1)).

Some authors only reported a difference score between prestimulation and poststimulation measures. In this case, SD from prestimulation, the mean was not provided and the SMD was calculated using the following Gibbons' equation [40]:

(2) $$\begin{array}{c} {SMD}_{Gibbons\left(G\right)}=c\left(n-1\right)\frac{{mean}_{difference}}{standard\;{deviation}_{difference}}, \end{array}$$

where *n* is the number of participants in the study and *c*(*n* − 1) = 1 − (3/(4(*n* − 1) − 1)).

### 2.4. Quality Assessment

For the methodological quality appraisal of the included studies, a standard quality assessment was used to evaluate original research studies [41]. Rating of individual studies was performed independently by two of the authors, and any discrepancy was resolved by a consensual decision. The summary score for each paper was expressed as the percentage of the potential maximum score.

Pre-consensus interrated agreement for each question on the quality assessment tool and for the score of each study was calculated with the Cohen's weighted kappa score [42]. The agreement between the two assessors was qualitatively interpreted in accordance with standardised recommendations [43].

## 3. Results

### 3.1. Literature Search Results

**Figure 1:**
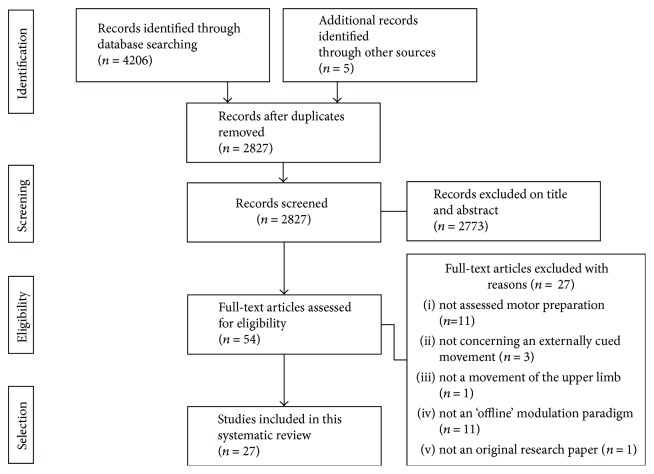
Flowchart of the systematic research strategy.

As shown in Figure 1, the primary systematic search strategy yielded 4206 references and 5 additional records identified through other sources. After discarding duplicates, 2827 titles and abstracts were screened and 2773 were excluded. Forty-nine potentially relevant full texts were reviewed, and 27 were rejected because they were not assessing motor preparation (*n* = 11), using an externally cued movement reaction time paradigm (*n* = 3) in the upper limb (*n* = 1) or using an offline neuromodulation paradigm (*n* = 11). Another study was excluded because it was not a research paper (*n* = 1). Twenty-seven studies met all the criteria and were included in this systematic review. It should be noted that several studies targeted more than one brain area or used several stimulation protocols. In such cases, each brain target or stimulation protocol was considered a separate study in Results.

### 3.2. Methodological Quality Assessment

Interrater pre-consensus agreement concerning the quality of the included studies was good according to the weighted Cohen's kappa score for the global score (*k* = 0.77, SD = 0.11; ranging from 0.6 to 1; see Supplementary Table 1), as well as for each of the fourteen quality assessment criteria (*k* = 0.76, SD = 0.16; ranging from 0.43 to 1; see Supplementary Table 2). The only criteria for which a fair level of agreement was obtained were regarding random allocation and analytic methods. After consensus between the two assessors, the average score for the methodological quality of the 27 included studies was 77% (SD = 11, ranging from 46.2% to 89.3%).

### 3.3. Study Characteristics

#### 3.3.1. Noninvasive Brain Stimulation Protocols

Of the 27 included studies, 14 used rTMS techniques to induce a transient modulation of a given cortical area using either high-frequency rTMS [44–46], low-frequency rTMS [47–54], or both [55–57]. Patterned rTMS was also used in 6 studies using cTBS [31, 58–61] or both cTBS and iTBS [62]. Finally, 7 studies used tDCS [63–69].

#### 3.3.2. Experimental Design

Twenty studies used a crossover design, 2 studies a parallel design, and 5 studies both crossover and parallel designs. Sham-controlled condition was used in 14 studies. For rTMS studies, sham condition was applied on a control site not involved in the motor preparation [48, 51, 52, 59] or at the same experimental stimulation position with the coil angled at approximately 45° from the scalp [44, 45, 50, 54, 55], to mimic sensation and noise artifacts of TMS without depolarizing cortical neurons [70]. For tDCS studies, five studies used a single-blind or double-blind sham condition consisting in applying 1 mA current (<30 s ramp-up time) then shutting off the current for the rest of the stimulation period, to generate similar scalp sensations than during the first seconds of stimulation without modulating neural activity [63–66].

### 3.4. Effect of Noninvasive Brain Stimulation over Dorsolateral Prefrontal Cortex (DLPFC) on Motor Preparation

**Figure 2:**
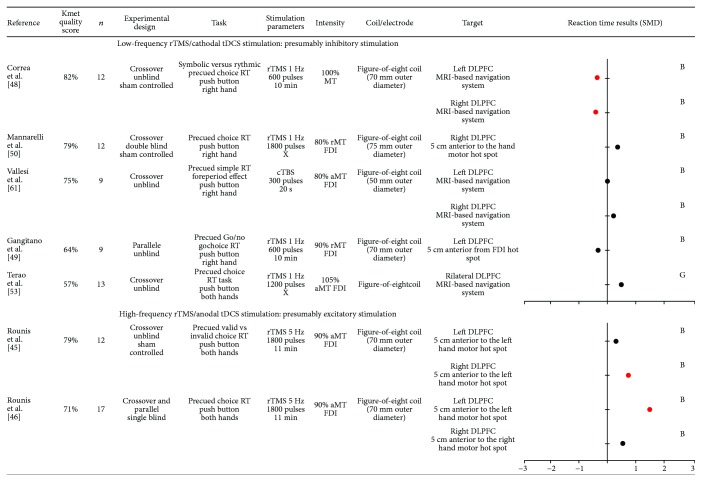
Characteristics of included studies that evaluated the role of dorsolateral prefrontal cortex (DLPFC) and results obtained on reaction time (RT) expressed in standard mean difference (SMD). Negative SMD values correspond to a decrease in RT after stimulation whereas positive SMD values correspond to an increase in RT after stimulation. Studies reported significant changes in RT after stimulation are illustrated by a red dot. The size of each dot depends on the sample size of the study. B = SMD Becker's equation; G = SMD Gibbons' equation; rTMS = repetitive transcranial magnetic stimulation; tDCS = transcranial direct current stimulation; MSO = maximal output stimulator; aMT = active motor threshold; FDI = first dorsal interosseous; MRI = magnetic resonance imagery.

Eleven studies investigating the effects of noninvasive brain stimulation over the DLPFC on motor preparation were retrieved (see Figure 2). Five studies using presumably inhibitory stimulation (1 Hz rTMS or cTBS) failed to reveal delayed RT after stimulation. Paradoxically, in two subgroups of studies using 1 Hz stimulation over both the right and the left DLPFC at different sessions, RT was found to be decreased but only for valid and symbolic warning signal [48]. A study investigating 5 Hz rTMS stimulation applied separately over the right and left DLPFC revealed that rTMS over the right DLPFC increased RT only for invalid trials whereas no effect was found for the left DLPFC [45]. Rounis et al. [46] also provided evidence of the role of the left DLPFC in attentional processes that occur during motor preparation. After applying 5 Hz rTMS, RT obtained after an invalid warning signal significantly increased in the motor attention task whereas no effect was found for the spatial attention task [46].

Overall, results obtained for the DLPFC are difficult to generalize because effects on RT appear to be very dependent on the paradigm used.

### 3.5. Effect of Noninvasive Brain Stimulation over Posterior Parietal Cortex (PPC) on Motor Preparation

**Figure 3 fig3:**
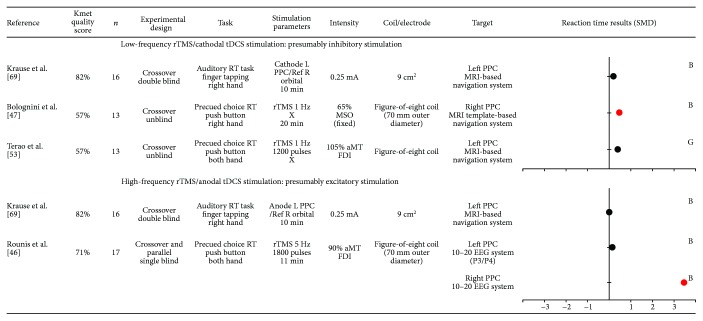
Characteristics of included studies that evaluated the role of posterior parietal cortex (PPC) and results obtained on reaction time (RT), expressed in standard mean difference (SMD). Negative SMD values correspond to a decrease in reaction time after stimulation whereas positive SMD values correspond to an increase in reaction time after stimulation. Studies reported significant changes in RT after stimulation are illustrated by a red dot. The size of each dot depends on the sample size of the study. B = SMD Becker's equation; G = SMD Gibbons' equation; Ref = reference; L = left; R = right; rTMS = repetitive transcranial magnetic stimulation; tDCS = transcranial direct current stimulation; MSO = maximal output stimulator; aMT = active motor threshold; FDI = first dorsal interosseous; MRI = magnetic resonance imagery; EEG = electroencephalography.

Six studies examined the role of the PPC during motor preparation (see Figure 3).

In a high-quality study, cathodal tDCS applied over the left PPC did not affect RT in a simple precued RT paradigm [69]. The role of the right PPC was addressed using a modality-specific (auditory or visual) or cross-modality (auditory and visual) choice precued RT paradigm. After 1 Hz rTMS, results indicated that RT increased when the warning signal was only auditory or visual, but not for combined audio-visual warning signal [47]. Another study using low-frequency rTMS over the PPC did not show an effect on RT in a precued choice RT [53].

Three studies using high-frequency rTMS or anodal tDCS over the PPC were retrieved. Anodal tDCS applied over the left PPC did not affect RT in a simple precued RT paradigm [69]. By delivering 5 Hz rTMS over the right PPC, RT increased for invalid trials presented in the left visual hemispace in a spatial attentional task. However, no significant changes on RT were observed after left PPC stimulation. Overall, the limited number of studies reporting a significant effect of stimulation on RT makes it difficult to conclude about the involvement of the PPC during motor preparation.

### 3.6. Effect of Noninvasive Brain Stimulation over Supplementary Motor Area (SMA) on Motor Preparation

**Figure 4 fig4:**
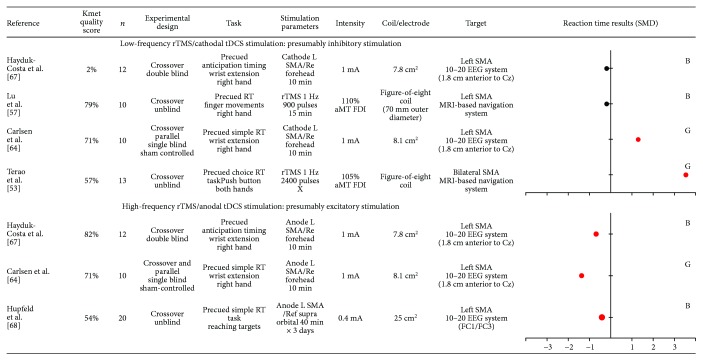
Characteristics of included studies that evaluated the role of supplementary motor area (SMA) and results obtained on reaction time (RT), expressed in standard mean difference (SMD). Negative SMD values correspond to a decrease in reaction time after stimulation whereas positive SMD values correspond to an increase in reaction time after stimulation. Studies reported significant changes in RT after stimulation are illustrated by a red dot. The size of each dot depends on the sample size of the study. B = SMD Becker's equation; G = SMD Gibbons' equation; Ref = reference; L = left; R = right; rTMS = repetitive transcranial magnetic stimulation; tDCS = transcranial direct current stimulation; MSO = maximal output stimulator; aMT = active motor threshold; FDI = first dorsal interosseous; EEG = electroencephalography; MRI = magnetic resonance imagery.

Systematic research retrieved seven studies that investigated the role of the SMA on motor preparation using rTMS (*n* = 2) or tDCS (*n* = 5) (see Figure 4).

Two studies demonstrated that low-frequency rTMS applied over the SMA increased RT in a simple RT paradigm [53, 64]. More specifically, results of Terao et al. [53] showed that stimulation induced a greater increase in RT when the warning signal delivered full information concerning the forthcoming movement, in comparison with the no-information condition. That suggests a specific role of the SMA during an advanced stage of motor preparation, when information concerning the forthcoming movement (like effector or target) is known to the participant. However, two high-quality studies reported no effect of presumably inhibitory stimulation, using either cathodal tDCS [67] or 1 Hz rTMS [57] over the SMA.

The three studies using high-frequency rTMS or anodal tDCS over the SMA demonstrated a reduction in RT in both simple and choice RT paradigms [64, 67, 68].

Overall, the majority of these findings indicate an implication of the SMA during motor preparation, with opposite patterns of results for presumably inhibitory (increased RT, although variable across studies) and presumably facilitatory (decreased RT) stimulation.

### 3.7. Effect of Noninvasive Brain Stimulation over Dorsal Premotor Cortex (PMd) on Motor Preparation

**Figure 5 fig5:**
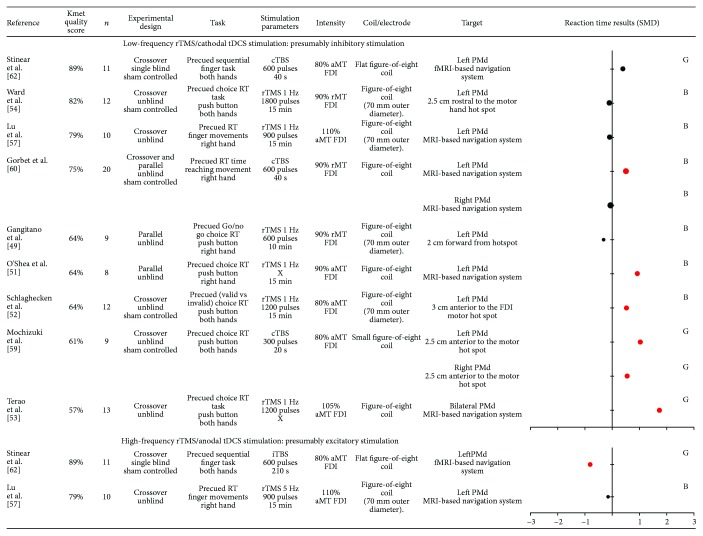
Characteristics of included studies that evaluated premotor dorsal cortex (PMd) and results obtained on reaction time (RT), expressed in standard mean difference (SMD). Negative SMD values correspond to a decrease in reaction time after stimulation whereas positive SMD values correspond to an increase in reaction time after stimulation. Studies reported significant changes in RT after stimulation are illustrated by a red dot. The size of each dot depends on the sample size of the study. B = SMD Becker's equation; G = SMD Gibbons' equation; rTMS = repetitive transcranial magnetic stimulation; TBS = theta burst stimulation; rMT = resting motor threshold; aMT = active motor threshold; FDI = first dorsal interosseous; APB = abductor pollicis brevis; MRI = magnetic resonance imagery.

On the 13 included studies examining the role of the PMd, 11 used high-frequency rTMS or anodal tDCS and 2 used low-frequency rTMS or cathodal tDCS over the PMd (see Figure 5).

A high-quality study using cTBS (presumably inhibitory) over the left PMd reported RT measured before the execution of a complex precued sequential finger movement to be unaffected [62]. In another high-quality study, Ward et al. [54] asked participants to perform a precued choice RT paradigm, with a button press response congruent with the direction indicated by a spatial cue warning signal. In some trials, the cue was invalid and participants had to update their motor goal depending on the response signal location. After transiently disrupting neuronal processing of the left PMd using 1 Hz rTMS, the authors observed a lower error rate but no difference in RT. In the same way, no difference on RT after low-frequency rTMS stimulation over the PMd was observed in two other studies [49, 57]. However, most of the included studies using presumably inhibitory stimulation found increased RT. Low-frequency rTMS applied over the left PMd, but not the right, increased RT in a precued reaching paradigm [60]. O'Shea et al. [51] found the same increase in RT immediately after low-frequency rTMS applied over the left PMd. Two other studies demonstrated that transient inhibition over the left PMd increased RT for movement performed with the contralateral hand [52, 53]. Finally, cTBS over both the left and the right PMd led to an increase in RT during a precued choice RT with arbitrary informative warning signal that indicates which hand to choose to press the button of the response signal apparition [59].

Using high-frequency rTMS stimulation over the left PMd, Stinear et al. [62] observed that RT decreased for complex sequences performed with the contralateral hand whereas Lu et al. [57] observed no change in RT in a similar paradigm.

Overall, despite some heterogeneity, the results support the involvement of the PMd during motor preparation and particularly in tasks requiring selection of action depending on visual-spatial arbitrary cues [51, 52, 60]. All studies reporting a significant change in RT support a reciprocal pattern of results for presumably inhibitory (increased RT) and presumably facilitatory (decreased RT) stimulation.

### 3.8. Effect of Noninvasive Brain Stimulation over Primary Motor Cortex (M1) on Motor Preparation

**Figure 6:**
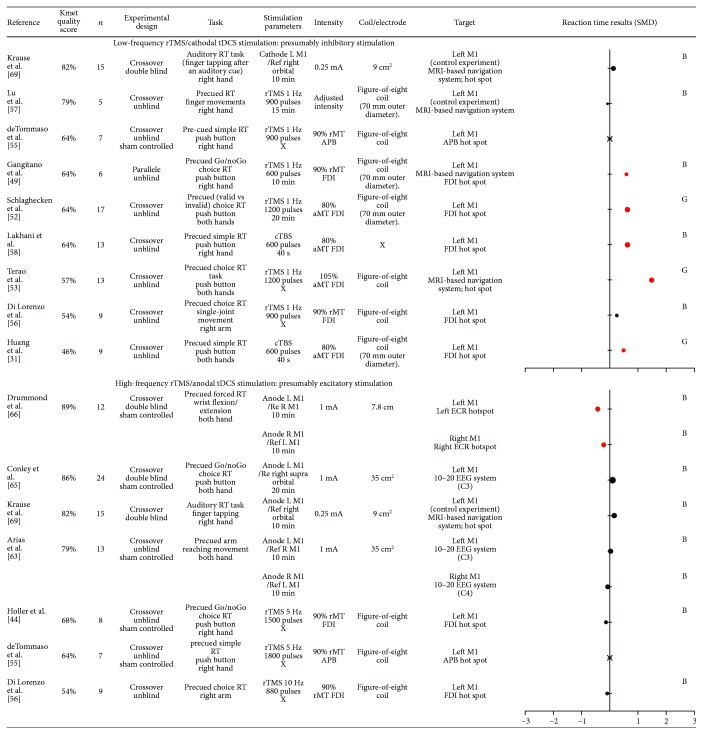
Characteristics of included studies that evaluated primary motor cortex (M1) and results obtained on reaction time, expressed in standard mean difference (SMD). Negative SMD values correspond to a decrease in reaction time (RT) after stimulation whereas positive SMD values correspond to an increase in reaction time after stimulation. Studies reported significant changes in RT after stimulation are illustrated by a red dot. The size of each dot depends on the sample size of the study. A cross indicates that data was not available. B = SMD Becker's equation; G = SMD Gibbons' equation; Ref = reference; L = left; R = right; rTMS = repetitive transcranial magnetic stimulation; TBS = theta burst stimulation; rMT = resting motor threshold; aMT = active motor threshold; FDI = first dorsal interosseous; APB = abductor pollicis brevis; MRI = magnetic resonance imagery; EEG = electroencephalography.

The M1 was the most frequently targeted area, with eighteen included studies delivering rTMS or tDCS over the left M1 contralateral to the upper limb performing the movement and one study delivering rTMS over the right and left M1 when the task involved both the right and the left hands (see Figure 6).

Four studies using presumably inhibitory stimulation over the M1 failed to reveal any effect on RT [55–57, 69]. However, five studies reported that low-frequency rTMS or cTBS increased RT in the contralateral hand [31, 49, 52, 53, 58].

Two high-quality studies applying anodal tDCS over the M1 reported no significant effect [65, 69]. Four others studies using either high-frequency rTMS or anodal tDCS applied over the M1 also failed to reveal any effect on RT [44, 55, 56, 63]. Only one recent study using anodal tDCS over the right and left M1 showed a decreased in a precued choice RT paradigm [66].

Taking together, the majority of the included studies demonstrates an impact of presumably inhibitory stimulation on RT and thus confirms the role of the M1 during motor preparation. However, presumably excitatory stimulation generally failed to reveal shorter RT, except for one study.

## 4. Discussion

**Figure 7:**
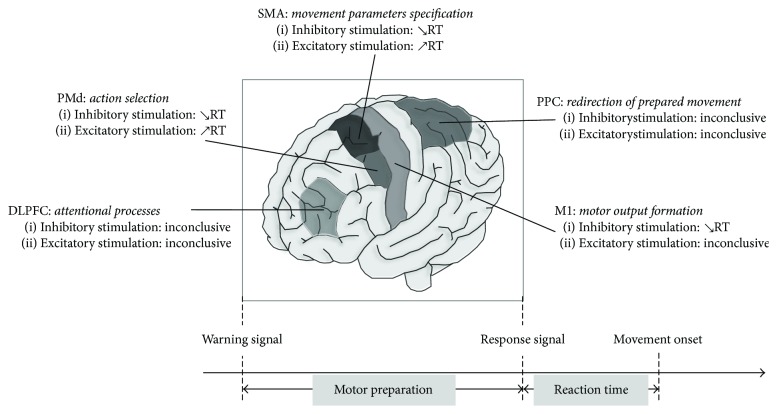
Neural correlates of motor preparation as investigated by noninvasive brain stimulation. DLPFC: dorsolateral prefrontal cortex; PMd: premotor dorsal cortex; SMA: supplementary motor area; M1: primary motor cortex; PPC: parietal posterior cortex.

This work is the first to systematically review findings from studies using noninvasive brain stimulation to investigate neural correlates of motor preparation. Findings were obtained from 27 studies with an overall good quality. The substantial methodological heterogeneity sometimes makes difficult direct comparisons between the studies. However, our results shed light on the functional implication of five cortical regions integrated into a frontoparietal network in various aspects of motor preparation: DLPFC, PPC, SMA, PMd, and M1 (see Figure 7).

### 4.1. Reaction Time: One Outcome for Multiple Motor Preparation Processes

Reaction time, which corresponds to the delay between the response signal appearance and motor response initiation, is the main outcome measure used to assess the efficiency of processes that occur during motor preparation [4]. However, RT is a global measure encompassing several underlying processes that can be decomposed into three independent components: (1) the first component, mainly attentional, reflects the time needed to create the motor goal and to make a choice if required by the task (i.e., choice RT task); (2) the second component reflects the time needed to encode perceptual and sensorimotor information to reach the motor goal (distance, amplitude, etc.); and (3) the third component reflects the time needed to initiate the motor action [1, 71]. Some RT paradigms tap more in one specific component than others. For example, paradigms including invalid or arbitrary warning signals focus more on the first component, while simple precued RT paradigms focus more on the third component. This highlights the importance of the choice of the RT paradigm in order to investigate the causal contribution of one particular brain region believed to play a specific role during motor preparation process and can explain some of the apparently discrepant results obtained in this systematic review. This will be discussed more in detail for each concerning brain area.

### 4.2. Neural Correlates of Motor Preparation

#### 4.2.1. Dorsolateral Prefrontal Cortex

Animal studies and neuroimaging studies in humans suggest that the DLPFC has a role in processing goal-relevant information and in alertness during motor preparation of voluntary movements [72, 73]. Results obtained for the DLPFC in this systematic review make it difficult to generalize its exact implication in motor preparation. Only two high-quality studies showed significant RT changes in specific precued paradigms using valid and invalid warning signals, designed to study attentional aspects during motor preparation [46, 48]. The lack of effect reported by several studies could be explained by the fact that DLPFC involvement is believed to take place only during early selection of the motor goal [74]. Therefore, the simple precued RT paradigm and the use of RT as an outcome variable might not be optimal to capture the implication of the DLPFC during motor preparation. For instance, one of the included studies showed that despite the absence of a change on RT, inhibitory rTMS significantly reduced the early period of CNV amplitude, known to reflect the perceptive and attentive processes that occur immediately after the warning signal apparition, when the motor goal has to be defined [50]. This result suggests that despite the lack of a clear effect of DLPFC stimulation on RT, this region appears to play a role in the allocation of attention to an intended motor action. Importantly, this highlights the fact that RT, although behaviorally relevant, is not necessarily sensitive to all aspects of motor preparation processes.

#### 4.2.2. Posterior Parietal Cortex

Previous studies using single-pulse TMS or fMRI during precued double-step paradigms, in which the target to reach is unpredictably displaced at the beginning of the movement initiation, have demonstrated that the PPC is critically involved in the redirection of an already prepared movement [75, 76]. Nevertheless, results of the present study concerning the PPC failed to show consistent evidence of a functional implication during motor preparation. The only study showing a decrease in RT after high-frequency rTMS used trials with invalid warning signals (i.e., trials in which the participants had to perform movements in a direction that differed from that indicated by the warning signal [46]). This suggests that the simple precued RT paradigm used in the other included studies was not optimal to demonstrate the implication of the PPC during motor preparation.

#### 4.2.3. Supplementary Motor Area

Neuroimaging and animal studies have suggested that the SMA is activated before voluntary movement execution [77, 78]. Findings obtained with both high-frequency rTMS or anodal tDCS and low-frequency rTMS or anodal tDCS over the SMA support a contribution of the SMA to motor preparation [53, 64, 68]. Results suggest that the SMA is particularly involved in an advanced stage of motor preparation (when the information concerning the forthcoming movement (effector or target) is known) which is coherent with previous neuroimaging studies (for a review, see [18]). An important point arising from neuroimaging studies that needs to be considered is that the SMA appears to be more involved during the preparation of self-initiated movements compared to externally cued movements (for a review, see [79]). Therefore, the fact that this systematic review was focusing only on externally cued movements probably explains why a small number of studies modulating SMA were retrieved. One of the included studies provides support for that view [57]. Two forms of movement-related cortical potentials were compared: the CNV that occurs during preparation of externally cued movement and another negative cortical potential called the Bereitschaftspotential (BP) that occurs about 1.5 s prior to a self-initiated movement production [80, 81]. Lu et al. [57] demonstrated that the CNV was unaffected by 1 Hz rTMS applied over the SMA whereas the late component of BP was increased.

#### 4.2.4. Dorsal Premotor Cortex

Previous neuroimaging studies reported that the PMd is activated during arbitrary instructing cues that allow making a choice about the forthcoming movement [14]. Moreover, it has been previously suggested that action selection processes that occur in the PMd are predominantly lateralized to the left hemisphere for right-handed participants [82, 83]. Overall, findings of this review support the involvement of the PMd in motor preparation, with a predominant implication of the left PMd [53, 60] in the selection of the forthcoming action during motor preparation [51, 52, 59, 60, 62]. Consistent with this view, Mochizuki et al. reported that pairs of single-pulse TMS applied over the PMd during motor preparation increased RT in choice but not in simple RT tasks [59]. Results of the review also confirmed that the PMd seems particularly involved when associations between arbitrary warning signal and specific motor responses are required (e.g., a large square or a small circle = index finger [51]) or when the warning signal delivers visuospatial information (e.g., a right or left arrow [60]). These findings are congruent with previous animal studies showing that specific PMd neurons increase their discharge rate when a particular spatial warning signal is presented to the monkeys [84].

#### 4.2.5. Primary Motor Cortex

Animal and single-pulse TMS studies provided evidence that the M1 is involved in motor preparation (for a review, see [6, 85]). Increased RT after M1 low-frequency rTMS or cTBS (presumably inhibitory) confirmed the role of the M1 during motor preparation [31, 49, 52, 53, 58], despite the fact that only one recent high-quality study reported shorter RT after anodal tDCS applied over the left and right M1 [66]. In addition to these changes in RT, two studies also measuring movement-related cortical potentials [44, 55] showed an increase in the late component of CNV. This effect, specifically on the late component, is consistent with the presumed role of the M1 in the preparation of the forthcoming motor response, rather than attentional or cognitive aspects of motor preparation [44, 86].

### 4.3. Methodological Consideration about Noninvasive Brain Stimulation

Two methods of noninvasive brain stimulation have been used in the included studies: repetitive TMS and tDCS. Repetitive TMS after-effects depend on the stimulation parameters (frequency and duration), and the basic mechanisms underlying this induced plasticity are probably mediated by changes in the synaptic transmission. Particularly, it has been proposed that rTMS after-effects share some features with long-term potentiation (LTP) by increasing the strength of excitatory synapses and long-term depression (LTD) by conversely altering the efficacy of the excitatory synapses [87–89]. Physiological mechanisms of tDCS-induced plasticity are quite different, mainly because the weak current passing between the anode and the cathode does not induce action potentials in axons, but instead modifies the average level of discharge in neurons [90]. Thus, during stimulation, the neuronal resting membrane potential is depolarized under the anodal electrode and hyperpolarized under the cathodal stimulation [91].

Studies included in this systematic review rely on the a priori hypothesis that specific stimulation paradigms transiently inhibit or excite cortical activity in the targeted area. This commonly assumed hypothesis is based on previous studies measuring corticospinal excitability changes at rest following stimulation over the M1. However, some studies bring evidence that this is not always simple (for a review, see [92]) with high intraindividual variability in MEPs changes after stimulation [92, 93]. In a recent position paper about noninvasive stimulations, authors estimate that the probability of producing the “expected” response as measured by MEPs may be lower than 50% [32]. Only a minority of the included studies reported stimulation after-effect on corticospinal excitability, even for studies targeting the M1. Importantly, changes in corticospinal excitability and in RT can sometimes be related, but can also occur independently from each other. For instance, one study reported changes in RT after cTBS only for participants who presented a decrease in MEPs amplitude measured at rest after stimulation [58], while another study demonstrated that cTBS applied over the left PMd decreased M1 corticospinal excitability in the contralateral hemisphere but without affecting RT [62]. Moreover, it needs to be kept in mind that while tDCS stimulation has been described as cathodal or anodal at the targeted cortical area, an effect on the cortical area located underneath the other electrode cannot necessarily be excluded (see Figures 2 [Fig fig3] [Fig fig4] [Fig fig5]–6 for precise electrode montage). Another potential limitation to keep in mind is the fact that several factors have been shown to impact the after-effects of transcranial stimulation such as the delay between the cessation of the stimulation and the onset of the testing (for a review see, [93]). This delay was highly variable (or not systematically reported) across the included studies, which might contribute to explain the heterogeneous results. Another aspect to consider is the fact that noninvasive brain stimulation can impact the neural network dynamic beyond the targeted cortical area, via direct neural interconnections or compensatory mechanisms [80, 81]. In order to better understand the effects at the network level, rTMS and tDCS can be combined with neuroimaging methods (for a review, see [94, 95]). For example, the study of O'Shea et al. [51] provides evidence that low-frequency rTMS applied over the left PMd was associated with a compensatory increase activity in the contralateral PMd, which probably explains why a preserved behavioral performance was observed. Globally, these methodological considerations may have contributed to the heterogeneity in the results included in this systematic review.

## 5. Conclusion and Future Perspectives

This systematic review demonstrates that noninvasive brain stimulation techniques can improve and/or disrupt motor preparation as characterized by RT changes in healthy participants. Since motor preparation includes many components from perceptive and attentional aspects to the motor command specification [1], different precued RT paradigms have been used and some appear to be more appropriate to capture the role of specific cortical areas of interest (e.g., simple or choice RT, with or without invalid warning signal). Moreover, while RT change is a relevant behavioral index of motor preparation, a combination of neuroimaging techniques like EEG or fMRI allows a better understanding of the effect of noninvasive brain stimulation at the network level. Finally, this review focused on RT changes that occur immediately after a unique session of stimulation in healthy participants. Cumulative sessions of stimulation applied in order to modulate the activity of the frontoparietal network may have a clinical potential in patients who presented an alteration of motor preparation [96, 97].

## Supplementary Materials

Supplementary Materials Table 1: interrater agreement before consensus for each question of the Kmet checklist. CI = confidence intervals; SD = standard deviation. Table 2: total Kmet score for each included study and interrater score pre-consensus. CI = confidence intervals; SD = standard deviation.
