# Supplementary material for: Stimulating the Healthy Brain to Investigate Neural Correlates of Motor Preparation: A Systematic Review
Source: Neural Plast. 2018 Feb 4;2018:5846096. doi: 10.1155/2018/5846096 (PMC5835236; doi:10.1155/2018/5846096)
Supplement: Supplementary Materials — Table 1: interrater agreement before consensus for each question of the Kmet checklist. CI = confidence intervals; SD = standard deviation. Table 2: total Kmet score for each included study and interrater score pre-consensus. CI = confidence intervals; SD = standard deviation. [file 5846096.f1.docx]

# Supplementary data

**Search strategy**

**Pubmed (n=23)**

(Preparation[Title/Abstract] OR activity[Title/Abstract] OR "motor planning"[Title/Abstract] OR "pre-motor planning"[Title/Abstract] OR movement[Title/Abstract] OR attention[Title/Abstract] OR alertness[Title/Abstract]) AND ((("contingent negative variation"[Title/Abstract] OR CNV[Title/Abstract] OR "reaction time"[Title/Abstract] OR "reaction response"[Title/Abstract] OR "response readiness"[Title/Abstract] OR "movement potential"[Title/Abstract] OR "pre motor potential"[Title/Abstract] OR "pre-motor potential"[Title/Abstract]) AND (tDCS[Title/Abstract] OR "Transcranial direct current"[Title/Abstract] OR "Transcraneal direct current"[Title/Abstract] OR "Repetitive transcranial magnetic"[Title/Abstract] OR "Repetitive transcraneal magnetic"[Title/Abstract] OR rTMS[Title/Abstract] OR "Theta Burst"[Title/Abstract] OR cTBS[Title/Abstract] OR "Neural activation"[Title/Abstract] OR TMS[Title/Abstract] OR neuromodulation[Title/Abstract] OR "noninvasive brain"[Title/Abstract] OR "non invasive brain"[Title/Abstract]) AND stimulation[Title/Abstract])) NOT (((mice OR mouse OR rat OR rats OR dog OR dogs OR cat OR cats OR rabbit* OR chick* OR sheep* OR monkey)))

**Medline (Ovid) (n=1765)**

(Preparation OR activity OR "motor planning" OR "pre-motor planning" OR movement OR attention OR alertness).ab,ti AND Reaction Time/ OR contingent negative variation/ OR evoked potentials, motor/ OR ((contingent adj1 negative) or CNV or (reaction adj1 (time or response or readiness)) or (potential adj1 (movement or pre motor or premotor or readiness))).ab,ti.AND MH "Transcranial Direct Current Stimulation" OR MH "Transcranial Magnetic Stimulation") OR ((theta adj3 Burst) or tDCS or ((Transcran?al or anodal or cathocal) adj3 stimul*) or rTMS or cTBS or TMS or neuromodulation).ab,ti.NOT (mice or mouse or rat or rats or dog or dogs or cat or cats or rabbit* or chick* or sheep* or monkey*).ab,ti.

**Embase (Elsevier) (n=1598)**

(Preparation OR activity OR (motor NEAR/2 planning) OR movement OR attention OR alertness OR (response NEAR/2 readiness)):ab,ti AND 'evoked muscle response'/exp OR 'contingent negative variation'/exp OR 'reaction time'/exp OR (contingent NEAR/2 variation):ab,ti OR cnv:ab,ti OR (reaction NEAR/2 (time OR response)):ab,ti ((movement OR 'pre motor' OR readiness) NEAR/2 potential*):ab,ti ('transcranial direct current stimulation' OR 'transcranial magnetic stimulation'/exp OR 'neuromodulation'/exp OR ((brain NEAR/2 stimulat*):ab,ti AND (noninvasive:ab,ti OR 'non invasive'):ab,ti)) OR tdcs:ab,ti OR ((transcran?al OR cathocal OR anodal) NEAR/3 stimulation):ab,ti OR rtms:ab,ti OR ctbs:ab,ti OR tms:ab,ti OR neuromodulation:ab,ti OR (theta NEAR/2 burst):ab,ti NOT animal*:ab,ti OR cats:ab,ti OR cat:ab,ti OR cattle:ab,ti AND chick*:ab,ti OR dogs:ab,ti OR dog:ab,ti OR goat*:ab,ti OR 'guinea pig':ab,ti OR cricetinae:ab,ti OR hamster*:ab,ti OR horse*:ab,ti OR mice:ab,ti OR mouse:ab,ti OR rabbit:ab,ti OR rat:ab,ti OR rats:ab,ti OR sheep*:ab,ti OR swine*:ab,ti

**PsycINFO (n=164)**

Title: (tDCS OR rTMS OR cTBS OR neuromodulation OR ((Transcranial OR transcraneal) NEAR/3 Stimulation) OR ((Cathodal OR anodal) NEAR/3 stimulation) OR "Theta Burst" OR ((brain NEAR/2 stimulat*) AND (noninvasive OR "non invasive"))) OR Abstract: (tDCS OR rTMS OR cTBS OR ((Cathodal OR anodal) NEAR/3 stimulation) OR "Theta Burst" OR neuromodulation OR ((brain NEAR/2 stimulat*) AND (noninvasive OR "non invasive"))) OR Index Terms:("Transcranial Magnetic Stimulation"

AND Title: (Preparation OR activity OR (motor NEAR/2 planning) OR movement OR attention OR alertness OR (response NEAR/2 readiness)) OR Abstract: (Preparation OR activity OR (motor NEAR/2 planning) OR movement OR attention OR alertness OR (response NEAR/2 readiness))

AND (Titlec OR Abstract:((contingent NEAR/2 negative) or CNV or (reaction NEAR/2 (time or response or readiness)) or (potential NEAR/2(movement or "pre motor" or premotor or readiness)))) OR ((Index Terms:("Reaction Time"))) OR ((Index Terms:("Contingent Negative Variation")))

NOT Title : (simulation or mice or mouse or rat or rats or dog or dogs or cat or cats or rabbit* or chick* or sheep* or monkey*).

**Web of science (n=656)**

TOPIC : (((transcranial OR transcraneal) NEAR/3 stimulation) OR (tDCS) OR (rTMS) OR (cTBS) OR ((Cathodal OR anodal) NEAR/3 stimulation) OR (Theta NEAR/3 burst) OR neuromodulation OR ((brain NEAR/2 stimulat*) AND (noninvasive OR "non invasive")))AND TOPIC : (Preparation OR activity OR (motor NEAR/2 planning) OR movement OR attention OR alertness OR (response NEAR/2 readiness)) AND TOPIC : (contingent NEAR/2 negative) or CNV or (reaction NEAR/2 (time or response or readiness)) or (potential NEAR/2 (movement or "pre motor" or premotor or readiness)) NOT TOPIC : (simulation or mice or mouse or rat or rats or dog or dogs or cat or cats or rabbit* or chick* or sheep* or monkey

| **Question** | **Criteria** | **Cohen's weighted**  **Kappa score  [95 % CI]** | | **Agreement** |
| --- | --- | --- | --- | --- |
| 1 | Question / objective sufficiently described? | 1 | [1-1] | Excellent |
| 2 | Study design evident and appropriate? | 0.83 | [0.78-1] | Very good |
| 3 | Method of subject/comparison group selection or source of information/input variables described and appropriate? | 0.63 | [0.29-1] | Good |
| 4 | Subject (and comparison group, if applicable) characteristics sufficiently described? | 0.79 | [0.52-1] | Very good |
| 5 | If interventional and random allocation was possible, was it described? | 0.44 | [0.18-0.70] | Fair |
| 6 | If interventional and blinding of investigators was possible, was it reported? | 0.91 | [0.63-1] | Very good |
| 7 | If interventional and blinding of subjects was possible, was it reported? | 0.65 | [0.39-0.91] | Good |
| 8 | Outcome and (if applicable) exposure measure(s) well defined and robust to measurement / misclassification bias? Means of assessment reported? | 0.89 | [0.56-1] | Very good |
| 9 | Sample size appropriate? | 0.70 | [0.37-1] | Good |
| 10 | Analytic methods described/justified and appropriate? | 0.43 | [0.17-0.70] | Fair |
| 11 | Some estimate of variance is reported for the main results? | 0.77 | [0.47-1] | Good |
| 12 | Controlled for confounding? | 0.84 | [0.50-1] | Very good |
| 13 | Results reported in sufficient detail? | 0.92 | [0.54-1] | Very good |
| 14 | Conclusions supported by the results? | 0.79 | [0.44-1] | Good |
| **Total** |  | **Mean = 0.76**  **SD = 0.16** | | **Good** |

**Table 1:** Inter-rater agreement before consensus for each question of the Kmet checklist.

CI = confident intervals; SD = standard deviation.

| **Studies** | **% Kmet** | **Cohen's weighted  Kappa score [95% CI]** | | **Agreement** |
| --- | --- | --- | --- | --- |
| Drummond et al. 2017 | 89.3 | 0.76 | [0,25-1] | Good |
| Stinear et al. 2009 | 89.3 | 0,7 | [0,28-1] | Good |
| Conley et al. 2015 | 85.7 | 0.65 | [0,13-1] | Good |
| Correa et al. 2014 | 82.1 | 0.65 | [0.25-1] | Good |
| Hayduk-Costa et al. 2013 | 82.1 | 0.84 | [0.53-1] | Very good |
| Krause et al. 2014 | 82.1 | 0.76 | [0.42-1] | Good |
| Ward et al. 2010 | 82.1 | 0.64 | [0.25-1] | Good |
| Arias et al. 2016 | 78.6 | 0.71 | [0.25-1] | Good |
| Lu et al. 2012 | 78.6 | 0.89 | [0.84-1] | Very good |
| Mannarelli et al. 2015 | 78.6 | 0.74 | [0.46-1] | Good |
| Rounis et al. 2006 | 78.6 | 0.74 | [0.36-1] | Good |
| Gorbet et al. 2011 | 75.0 | 0.74 | [0.50-0.99] | Good |
| Vallesi et al. 2007 | 75.0 | 0.74 | [0.41-1] | Good |
| Carlsen et al. 2015 | 71.4 | 1 | [1-1] | Excellent |
| Rounis et al. 2007 | 71.4 | 1 | [1-1] | Excellent |
| Holler et al. 2006 | 67.9 | 0.89 | [0.67-1] | Very good |
| de Tommaso et al. 2012 | 64.3 | 0.65 | [0.35-0.95] | Good |
| Gangitano et al. 2008 | 64.3 | 0.83 | [0.59-1] | Very good |
| O'Shea et al. 2007 | 64.3 | 0.91 | [0.75-1] | Very good |
| Schlaghecken et al. 2003 | 64.3 | 0.82 | [0.58-1] | Very good |
| Lakhani et al. 2014 | 63.6 | 0.62 | [0.30-0.95] | Good |
| Mochizuki et al. 2005 | 60.7 | 0.84 | [0.63-1] | Very good |
| Bolognini et al. 2009 | 57.1 | 0.91 | [0.74-1] | Very good |
| Terao et al. 2007 | 57.1 | 0.7 | [0.38-1] | Good |
| Di Lorenzo et al. 2012 | 57.1 | 0.9 | [0.71-1] | Very good |
| Hupfeld et al. 2017 | 53.6 | 0.77 | [0.39-1] | Good |
| Huang et al. 2005 | 46.2 | 0.6 | [0.21-1] | Fair |
| T**otal** | **Mean = 71.1**  **SD = 11.6** | **Mean = 0.77 SD = 0.11** | | **Good** |

**Table 2**: Total Kmet score for each included studies and inter-rater score pre-consensus. CI = confident intervals; SD = standard deviation.
